# The Autophagy Signaling Pathway: A Potential Multifunctional Therapeutic Target of Curcumin in Neurological and Neuromuscular Diseases

**DOI:** 10.3390/nu11081881

**Published:** 2019-08-13

**Authors:** Lorena Perrone, Tiziana Squillaro, Filomena Napolitano, Chiara Terracciano, Simone Sampaolo, Mariarosa Anna Beatrice Melone

**Affiliations:** 1 Department of Chemistry and Biology, University Grenoble Alpes, 2231 Rue de la Piscine, 38400 Saint-Martin-d’Hères, France; 2 Department of Advanced Medical and Surgical Sciences, 2nd Division of Neurology, Center for Rare Diseases and InterUniversity Center for Research in Neurosciences, University of Campania “Luigi Vanvitelli”, via Sergio Pansini, 5, 80131 Naples, Italy; 3 Sbarro Institute for Cancer Research and Molecular Medicine, Department of Biology, BioLife Building (015-00)1900 North 12th Street, Temple University, Philadelphia, PA 19122-6078, USA

**Keywords:** autophagy, curcumin, polyphenols, neurological diseases, neuromuscular diseases, mTOR, signaling pathway, therapeutic target

## Abstract

Autophagy is the major intracellular machinery for degrading proteins, lipids, polysaccharides, and organelles. This cellular process is essential for the maintenance of the correct cellular balance in both physiological and stress conditions. Because of its role in maintaining cellular homeostasis, dysregulation of autophagy leads to various disease manifestations, such as inflammation, metabolic alterations, aging, and neurodegeneration. A common feature of many neurologic and neuromuscular diseases is the alteration of the autophagy-lysosomal pathways. For this reason, autophagy is considered a target for the prevention and/or cure of these diseases. Dietary intake of polyphenols has been demonstrated to prevent/ameliorate several of these diseases. Thus, natural products that can modulate the autophagy machinery are considered a promising therapeutic strategy. In particular, curcumin, a phenolic compound widely used as a dietary supplement, exerts an important effect in modulating autophagy. Herein, we report on the current knowledge concerning the role of curcumin in modulating the autophagy machinery in various neurological and neuromuscular diseases as well as its role in restoring the autophagy molecular mechanism in several cell types that have different effects on the progression of neurological and neuromuscular disorders.

## 1. Introduction

Curcumin (diferuloylmethane; 1,7-bis(4-hydroxy-3-methoxyphenyl)hepta-1,6-diene-3,5-dione) is an active constituent derived from the powdered rhizome of Curcuma longa [1]. This natural product, which belongs to the polyphenols, is the spice (curry, turmeric) commonly used in Asian cuisine and represents a widely studied nutraceutical and the most popular dietary supplement in the world. Curcumin has attracted increasing scientific and clinical interest thanks to its wide range of beneficial functions, including its antioxidant, anti-inflammatory, antiproliferative, antitumor, analgesic, cholesterol-lowering, hemostatic, antidiabetic, and antiamyloid roles. Other beneficial functions include its cyto-, gastro-, and neuro-protective roles as well as antiviral and antibacterial functions [2,3,4,5]. Curcumin is particularly attractive as a potent therapeutic substance, being a non-mutagenic and non-genotoxic agent, although scarcely bioavailable because of its hydrophobic nature [6]. In general, in humans, curcumin is recognized as a safe bioactive compound, even if used in high doses [5]. Several studies suggest that curcumin, like other polyphenols, is a highly pleiotropic molecule that interacts simultaneously with a wide range of molecular targets and influences numerous biochemical and molecular cascades, modulating the activation of various transcription factors and thus regulating the expression of growth factors, receptor complexes, cytokines, and enzymes involving cell proliferation and apoptosis [1,7,8]. The beneficial effect of curcumin in modulating autophagy through various cell signals, such as PI3K/Akt/mTOR, AMPK, MAPK/ERK1/2, Bcl-2, and Rab GTPase network, has also been demonstrated [4]. Autophagy is a lysosomal catabolic mechanism critical in maintaining cellular homeostasis under both physiological and pathological conditions [9] and represents the process by which cells adapt their metabolism to conditions of environmental or intracellular stress [10]. The multi-step nature of autophagy causes its susceptibility to being damaged at different levels, and its defective activity has been linked to a variety of human diseases [11,12]. Over the years, increasing evidence has accumulated on the dysfunction of autophagy in neurological and neuromuscular diseases, showing how the dysregulation of autophagy initiation, autophagosome formation, maturation, and the autophagosome-lysosome fusion phase contribute to the pathogenesis of these disorders. Because of its numerous beneficial properties, curcumin has been used in a wide range of clinical studies as a drug or adjuvant in the treatment of diseases, including those characterized by defective autophagy [11]. Curcumin shows both activating [13] and inhibitory [4] actions on autophagy mechanisms. In particular, it regulates AMPK and can inhibit mTOR level/activity [14]. The exact molecular mechanism by which curcumin exerts its role as an autophagy modulator remains to be elucidated; however, its activity as a potential therapeutic agent appears to be essential in different human disorders. In this review, we first state the metabolic pathways of autophagy and the possible implications for neurological and neuromuscular diseases. We then summarize the diverse bioactivity and health benefits of curcumin. Finally, we discuss how curcumin targets autophagy-related pathways in neurological and neuromuscular diseases.

## 2. Autophagy: Mechanisms and Regulation

**Figure 1:**
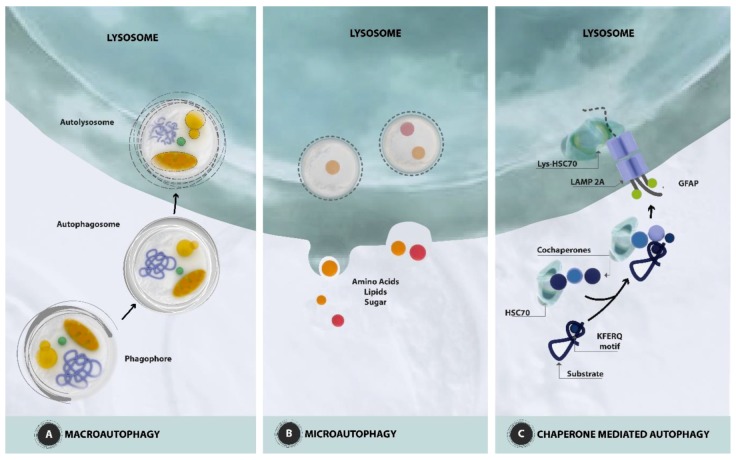
Schematic presentation of autophagic pathways. (**A**) Macroautophagy. The key event in macroautophagy is the *de novo* formation of a new organelle called the autophagosome, which surrounds and sequesters either random portions of the cytoplasm or selectively targets individual cytosolic components. Initially, double-membraned cup-shaped structures, called phagophores or membrane isolation, engulf the cytosolic cargo. The expansion of the double membrane ends with the completion of the autophagosome that is trafficked by microtubules. The fusion of the autophagosome with the lysosome constitutes an autolysosome where the trapped cargo can be degraded. (**B**) Microautophagy. The cytosolic cargo (amino acids, lipids, sugar) is translocated into the lysosomes for degradation via direct invagination, protrusion, or septation of the lysosomal limiting membrane. (**C**) Chaperone mediated autophagy. It selects a defined pool of proteins that contains the KFERQ motif and delivers them to lysosomes via the chaperone HSC70 and cochaperones. The complex formed by HSC70 and the KFERQ-containing protein interacts with the lysosome-associated surface membrane protein type 2A (LAMP2A). Then, the KFERQ-containing protein unfolds and is transported across the lysosome membrane by a lysosome form of HSC70 (lys-HSC70), which resides inside the lysosome. GFAP, glial fibrillary acidic protein.

Autophagy maintains the cellular proteostasis by degrading misfolded and long-lived proteins. It also removes damaged organelles [15]. It is divided into three subtypes according to the mechanism by which the intracellular material is destined to the lysosomes to be degraded: macroautophagy [16], microautophagy [17], and chaperone-mediated autophagy [18] (Figure 1).

These three subtypes show different mechanisms of cargo recognition and molecular support. However, all these subtypes have lysosomes as the final target for cargo digestion and product recycling. Microautophagy occurs when cytoplasmic material is introduced into lysosomes through direct invagination of the lysosomal membrane. Chaperone-mediated autophagy (CMA) is characterized by direct translocation into lysosomes for the degradation of proteins containing a defined pentapeptide pattern (KFERQ). Macroautophagy is characterized by the formation of double-membrane subcellular structures, called autophagosomes, which transport the degradable content from the cytoplasm and route them into lysosomes for degradation. Then, the cells reuse these products of degradation.

Macroautophagy initiates with the formation of the double membrane (phagophore), which is defined as nucleation. The phagophore derives from the plasma membrane, Golgi, endoplasmic reticulum or mitochondria [19], and envelops misfolded proteins or damaged organelles. The expansion of the phagophore ends with the completion of the autophagosome. The fusion of the autophagosome with lysosome constitutes an autolysosome within which the enclosed material, known as autophagic cargo, is degraded [20].

At the molecular level, macroautophagy is initiated by two major complexes: (a) the UN51-like Ser/Thr kinases (ULK) complex and (b) the class III phosphatidylinositol-3-kinase (PI3K) that are recruited to the phagophore assembly site (PAS) [21]. The ULK complex includes the ULK1/2 family, the FAK family kinase interacting protein of 200 kDa (FIP200), and ATG13 [22]. The PI3K complex, also defined as the Beclin1 complex, contains vacuolar protein sorting 34 (Vps34), p15 (VPS15), Beclin1 (ATG6), and Barkor (ATG14) [23]. Beclin1 localizes on the ER membrane and is regulated by the anti-apoptotic dimer BCL-2 and BCL-XL. When autophagy is activated, Beclin1 dissociates from the BCL-2 complex and coordinates with Vps34 [24]. Then, the bulk phosphatidylinositol 3-phosphate [PtdIns(3)P] is recruited on the surface of the phagophore [5]. Two ubiquitin-like complexes are responsible for the extension and closure of the autophagosome. The first complex is initiated by the interaction of Atg7 with Atg5, which, in turn, binds covalently to Atg12 [25]. This complex interacts with Atg16 to form the Atg5-Atg12-Atg16 complex, which elongates the phagophore. The interaction between Atg9 with Atg2 and Atg18 is responsible for the trafficking between the Trans-Golgi Network, endosomes, and newly formed autophagosomes. Atg4B cleaves to the microtubule-associated protein 1 light chain 3 (LC3) in another ubiquitin-like complex, leading to the formation of LC3-I [26]. Upon an autophagic signal, LC3-I is conjugated by Atg7, Atg3, and Atg12-Atg5-Atg16L multimers to a phosphatidylethanolamine (PE) moiety for the generation of the LC3-II form, which is considered a marker of autophagosome [27]. Next, dynein and other motor proteins are involved in the transport of autophagosomes along the microtubules [28]. Finally, the SNARE proteins (Soluble NSF Attachment Protein Receptors) are recruited on the lysosomes that fuse with the autophagosomes, leading to the degradation of the cargoes [29].

Autophagy is induced by both physiological conditions and stress, such as hypoxia and food deprivation [30]. Thus, different pathways regulate the autophagy machinery.

The IGF-1/Insulin pathways sense the nutrient variations and regulate growth, morphogenesis, and survival. In *C.elegans*, a link has been shown between the IGF-1/Insulin pathway and autophagy. Mutants in certain autophagy genes affect IGF-1/Insulin-induced cell growth [31]. In mammals, insulin withdrawal induces autophagic cell death in hippocampal neural stem cells [32]. IGF-1/insulin, along with the mammalian target of rapamycin (mTOR), regulates autophagy at several levels. mTOR is a negative regulator of autophagy, which is modulated by proteins acting upstream to mTOR signaling [33]. PTEN and TSC1/2 induce autophagy, whereas Akt inhibits it [33]. Elongation factor-2, a downstream effector of mTOR, also modulates autophagy [34]. The inhibitor of the mTOR complex 1 (MTORC1) induces the nuclear translocation of the transcription factor EB (TFEB) by phosphorylating key serine residues of TFEB, which in turn activates the expression of genes involved in autophagy and lysosomal biogenesis [35,36]. The thioredoxin interacting protein (TXNIP), which modulates the cell and body metabolism and is down-regulated by insulin [37,38,39], modulates autophagy through the mTOR pathway [40]. In addition, TXNIP suppresses the activity of Atg4B, leading to activation of the autophagic flux [41].

The tumor suppressor p53 modulates autophagy by regulating the expression of autophagy-related genes [34]. P53 promotes autophagy by inducing the expression of the damage-regulated autophagy modulator (DRAM), which is an integral lysosomal membrane protein and assists in the accumulation of autophagosomes [42]. P53 regulates autophagy by modulating the expression of chromatin-remodeling factors, such as e2f1 [43], which binds the gene regulatory regions of the autophagy proteins Atg1, Atg8, and DRAM [44].

The transcription factors FOXO directly regulate the expression of Atg8 and Atg12 [45]. In both normal growth and starvation conditions, Sirt1 promotes autophagy by acetylating Atg5, Atg7, and Atg8 [46]. Reactive Oxygen Species (ROS) are metabolites produced by several cellular activities, mostly by respiration, and induce oxidative stress. The majority of ROS production occurs in mitochondria. ROS are very reactive species, and their deleterious effects are counteracted by autophagic degradation of mitochondria (mitophagy) and anti-oxidant defense [47]. Indeed, ROS are necessary for the induction of autophagy and activate Atg4 [48]. Moreover, several stresses, such as hypoxia and exercise, induce ROS-dependent autophagy [49].

CMA differs from the other two types of autophagy because it selects a defined pool of proteins that contains the KFERQ motif and delivers them to lysosomes. This peptide sequence is recognized by the heat shock protein of 70 kDa (hsc70), which is the chaperone targeting these proteins to lysosomes [50]. The complex formed by hsc70 and the KFERQ-containing protein interacts with the lysosome-associated surface membrane protein type 2A (LAMP 2A). Then, the KFERQ-containing protein unfolds and is transported across the lysosome membrane by a lysosome form of hsc70 (lys-hsc70) [50].

Autophagy typically enhances cell viability. However, alterations in its regulation are implicated in the pathogenesis of neurological and neuromuscular diseases. Indeed, it seems that induction of autophagy at early stages has a protective function against neurotoxicity [51,52]. Because of its relevance in the pathogenesis of these diseases, it is the target of several pharmaceutical compounds. The characterization of natural compounds that target autophagy is an integral part of ongoing research aimed at finding therapeutic strategies for such diseases. In particular, recent studies emphasize the therapeutic challenges of curcumin in autophagy molecular mechanisms by highlighting curcumin as a potential therapeutic compound in neurological and neuromuscular diseases.

## 3. Curcumin Structure and Activity

**Figure 2:**
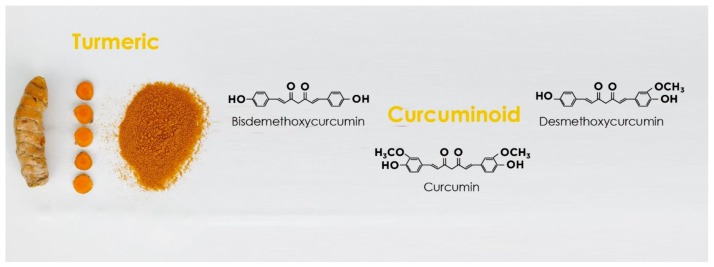
Curcuminoids in turmeric and their chemical structures.

Curcumin is a polyphenol derived from the turmeric root of Curcuma longa [1]. Curcumin possesses two similar aromatic rings in which the o-methoxy phenolic groups are linked to an α,β-unsaturated β-diketone moiety [53] (Figure 2).

Curcumin can also act as an electron donor in many redox reactions because it contains conjugated double bonds in its chemical structure, thereby stabilizing the structure [53]. Notably, curcumin at a low concentration can act as an antioxidant agent and counteract the formation of oxidative stress because it functions as a scavenger of ROS [54]. Oxidative stress is the result of an imbalance between the formation of ROS and the cellular antioxidant mechanisms, leading to peroxidation of membrane lipids and oxidative damage of proteins and DNA. Because of its structure, curcumin accumulates in hydrophobic regions, such as the plasma membrane, and decreases lipid peroxidation. In addition, curcumin stimulates antioxidant enzymes, such as catalase, superoxide dismutase (SOD), glutathione peroxidase (GPx), and heme oxygenase 1 (OH1), counteracting the oxidative damage [55]. On the contrary, at higher concentrations, curcumin shows a pro-oxidant activity that promotes cancer cell apoptosis, playing an important function in cell death during the neoplastic process [56]. Indeed, curcumin has a beneficial effect against cancer [57,58,59].

Concerning the effect of oxidative stress on inflammation, several studies have investigated the effect of curcumin as an anti-inflammatory agent. These studies confirmed that curcumin inhibits inflammation by blocking several pro-inflammatory molecule (such as cyclooxygenase 2 (COX2) and lipoxygenase 5 (LOX5)), inducible nitric oxide synthase (iNOS), inflammatory cytokines (such as tumor necrosis factor α (TNFα)), interleukin-(IL-) 1, 2, 6, 8, and 12, monocyte chemoattractant protein 1 (MCP1), and transcription factors (such as activating protein 1 (AP1), and nuclear factor κB (NF-κB) [59]. Thus, curcumin is believed to be beneficial against several diseases, including cancer, diabetes, and cardiovascular diseases [55,59] as well as neurodegenerative and neuromuscular diseases, which we describe later in this review.

Curcumin is considered a beneficial treatment for several diseases because it confers health benefits and is well tolerated without any toxicity at high oral doses. It has been shown that patients tolerate up to 2.2 g of Curcuma extract containing 180 mg of curcumin/day for four months [60]. Recently, we demonstrated that 1200 mg/day of curcumin for six months was well tolerated and reduced the size of tumors in neurofibromatosis type 1 patients [57].

Also, curcumin is proposed as a therapeutic agent for brain tumors and neurodegenerative diseases, since it can cross the blood-brain barrier (BBB) after oral administration when it is in its native form (unglucuronidated and unsulfated) [61].

Interestingly, curcumin is naturally fluorescent and binds misfolded proteins that are a pathogenic characteristic of several neurodegenerative diseases, such as the amyloid-beta aggregates in Alzheimer’s disease (AD) [62]. For this reason, curcumin has been used to label the amyloid plaques [62]. Moreover, curcumin can inhibit the aggregation of misfolded proteins and enhance their clearance [61]. This function will be discussed in more detail below in this review.

Despite the beneficial effect of curcumin for health and the cure and prevention of several diseases, its clinical application shows limitations due to curcumin’s poor bioavailability. Indeed, oral treatment is affected by curcumin’s slow water solubility, poor absorption, rapid metabolism, and systemic elimination. For this reason, there are several studies aimed at improving the bioavailability of curcumin using different approaches: the use of adjuvants, such as piperine, quercetin, and resveratrol [63]; nanoparticle-based delivery of curcumin using liposomes, solid lipid nanoparticles, niosomes, polymeric nanoparticles, polymeric micelles, cyclodextrins, dendrimers, and silver and gold nanoparticles [64,65]. In addition, synthetic structural analogs of curcumin have been developed in order to improve its bioavailability [66,67]. Interestingly, a recent study by our research group indicated that the bioavailability of curcumin significantly increases when its oral administration is associated with the Mediterranean Diet (MeDi) [57,68]. These data suggest that the high concentration of extra virgin olive oil polyphenols and/or fatty acids present in the MeDi contributes to increasing the bioavailability of curcumin and enhancing its effects [57].

## 4. Autophagy Modulation and the Interplay between Autophagy and Curcumin as a Therapeutic Approach for Neurological Disorders

Autophagy impairment is proved to have relationship with aging and neurodegenerative diseases. In a mouse model, very recently it has been shown that restoring autophagic flow attenuates neurodegeneration by promoting nuclear translocation of TFEB through inhibition of MTOR [69].

As described in the previous paragraphs, mTOR plays a critical role in both autophagic and lysosomal biogenesis through regulating TFEB and TFE3 nuclear-cytoplasmic shuttling. Therefore, targeting these processes is a prime strategy for developing therapies for different neurodegenerative diseases. Currently known TFEB activators are mainly MTOR inhibitors. However, recent experimental evidences suggest that new autophagy enhancers can act through both the MTOR-dependent and the MTOR-independent pathways, representing potential therapeutic agents for the treatment of neurodegenerative diseases. Intriguingly a recent article by Ju-Xian Song et al. demonstrated that a new curcumin analogue binds and activates TFEB in vitro and in vivo, independently of MTOR inhibition [70].

**Figure 3:**
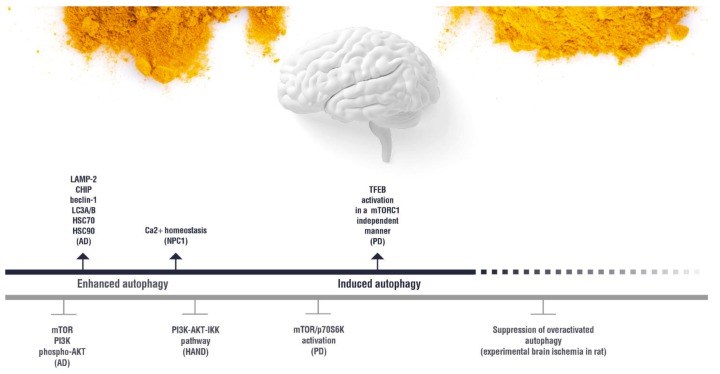
Overview of curcumin effects on the autophagy pathways in neurological diseases. Biological effect of curcumin on the molecules involved in the autophagy regulation in the neurological diseases discussed in the present review. 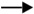 (induction); 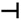 (inhibition). AD: Alzheimer’s disease; HAND: HIV-induced neurocognitive disorder; PD: Parkinson’s disease; NPC1: Niemann Pick C1.

In this paragraph, we discuss the effect of curcumin on the autophagy-lysosomal pathways in nervous system (CNS) disorders. Compared to a poor bioavailability and stability of curcumin in vivo, its amphiphilic nature allows its absorption, bioavailability, and half-life profiles to be very favorable in the CNS, due to the huge amounts of lipids that the brain contains [61]. To better understand the therapeutic potential of curcumin in CNS diseases, we have selected from the most recent literature those neurological diseases in which there is *in vitro* and *in vivo* evidence of the effectiveness of curcumin in contrasting, stopping, or reversing the cascade of pathogenic events through molecular interactions on the autophagic pathways (Figure 3).

### 4.1. Neurodegenerative Disorders

Alzheimer Disease (AD) is clinically characterized by progressively worsening memory loss, cognitive dysfunction, and changes in behavior and personality [71]. Widespread brain [70] accumulation of amyloid plaques and neurofibrillary tangles, which prevails in definite cerebral structures, is the pathological hallmark of AD [71]. The amyloid plaques are constituted by misfolded amyloid β (Aβ) peptides which derives from the proteolysis of the amyloid precursor protein (APP) [69]. The Aβ forms toxic oligomers that induce synaptic dysfunction [71,72,73]. Indeed, mutant APP leads to familial AD [71,74]. The neurofibrillary tangles are formed by hyper-phosphorylated microtubule-associated protein tau [71,75]. In AD patients, the first molecular and cellular alterations occur decades before the diagnosis of the disease. Risk and environmental factors, including nutrition, play a key pathogenic role in AD [39,71,76]. However, at present, there are neither efficient therapies nor an early diagnosis for AD.

The accumulation of misfolded Aβ and hyper-phosphorylated tau is a pathological hallmark of AD. The accumulation of these misfolded aggregates leads to synaptic dysfunction and neurodegeneration. Thus, the degradation pathways play a key role in the maintenance of the neuronal function. Among all the protein degradation systems, the molecular chaperones and autophagy play vital roles in the degradation of misfolded protein aggregates [77]. However, the activity of these systems is affected in AD, promoting the progression of this disease [78]. For this reason, compounds capable of restoring autophagy represent a therapeutic strategy. Several studies demonstrate that curcumin exerts a beneficial effect in AD. We will only mention the fact that curcumin inhibits the formation of Aβ oligomers and fibrils from the Aβ monomer; it also destabilizes the already-present Aβ fibrils, blocking Aβ-induced neurotoxicity [61,79,80]. Curcumin also prevents Aβ toxicity by binding the redox-active metals iron and copper [81], which enhance Aβ aggregation [73].

Animal models of AD treated with curcumin show enhanced autophagy, detected by LC3 immunofluorescence and protein content by western blotting [82]. This study demonstrates that curcumin lowers the protein content of Phosphatidylinositol 3-Kinase (PI3K), phosphorylated Akt, and the inhibitor of autophagy, mTOR, leading to enhanced autophagy [82]. Other studies have revealed that monocytes from AD patients pretreated with curcumin displayed inhibition of miR-128, leading to enhanced Aβ(1–42) degradation [83,84]. Maiti and colleagues demonstrated that dietary curcumin and solid lipid particles driving curcumin absorption (SLCP) induce autophagy in human (SH-SY5Y) and mice (N2a) neuronal cells treated with the Aβ(1–42) [85]. These authors showed that curcumin and SLCP increase the protein level of hsc70 and hsc90 in neuronal cells treated with the amyloid beta peptide, enhancing CMA [85]. Curcumin and SLCP also counteract the alterations induced by Aβ on the protein level of the autophagic proteins LAMP-2, CHIP, Beclin-1, and LC3I/II in neuronal cells in vitro [85]. Using a SH-SY5Y neuronal cell line treated with paraquat as an in vitro model of neurotoxicity, researchers have shown that pre-treatment with curcumin results in a significant decrement of APP expression and protein production [86]. Notably, in this in vitro model, pre-treatment with curcumin has the beneficial effect of increasing autophagy by restoring the protein level of LC3I/II, which is affected by paraquat in the absence of curcumin [86]. Also, curcumin induced the heat shock proteins involved in CMA and reduced tau pathology in vivo in a human tau mouse model [87]. On the other hand, Zhang and colleagues showed that curcumin had a beneficial effect on the viability of a mouse hippocampal neuronal cell line HT-22 treated with Aβ(1–42), and this beneficial effect parallels the reduction of autophagosomes, detected by transmission electron microscopy [88].

Parkinson’s Disease (PD) is characterized by selective neurodegeneration of dopaminergic neurons, mostly in the frontal lobe and striatum, and by the presence of Levy bodies, which are mostly composed by α-synuclein (α-syn) [89]. In particular, aggregation of α-syn plays a crucial role in PD pathogenesis [89]. Mutations in α-syn, such as the mutant A53T, increase its aggregation rate and are characteristic of familial PD [89]. Curcumin binds α-syn and blocks its aggregations; it also inhibits the aggregation of Aβ (see above). Autophagy is impaired in patients with dementia with Levy bodies and transgenic mice carrying mutant α-syn [90]. Induction of autophagy promotes α-syn degradation [91]. It has been shown that curcumin blocks the cell toxicity induced by α-syn in vitro [92]. In a cellular model of PD, the mutant A53T α-syn expressed in an SH-SY5Y neuronal cell line leads to a decrement of autophagy, detected by observing the decrement of the LC3-GFP punctate formation [93]. In addition, LC3-GFP punctate did not co-localize with α-syn, suggesting a poor degradation of the latest protein via autophagic flux [93]. In agreement, A53T α-syn expression resulted in a strong reduction of the LC3 II protein and enhanced phosphorylation of mTOR and its downstream effector, p70S6K, which are inhibitors of the autophagic flux [93]. Treatment with curcumin abolishes A53T α-syn-induced activation of the mTOR/p70S6K pathway, restoring autophagy and lowering the accumulation of A53T α-syn [93]. Thus, curcumin has a beneficial effect on α-syn degradation by blocking the autophagy inhibitor mTOR. The curcumin-induced α-syn degradation results in a beneficial effect on neuronal cells by blocking the cytoskeletal pathology induced by A53T α-syn [93]. The synthetic analog of curcumin named C1 activates autophagy by inducing the activation of TFEB in an MTORC1-independent manner in vitro and in vivo, without any change in TFEB serine phosphorylation, through direct binding to TFEB. Such TFEB-C1 interaction inhibits the association between TFEB and YWHA, which occurs after MTORC1-induced TFEB serine phosphorylation, and such TFEB-YWHA is sequestered in the cytoplasm [70]. Thus, C1-induced TFEB nuclear translocation promotes the autophagic flux and lysosome biogenesis both in cell culture in vitro and the rat brain in vivo in the frontal cortex and striatum [70].

Niemann Pick C1 (NPC1). NPC1 belongs to a larger family of diseases called lysosomal storage diseases (LSDs), which consist of the progressive accumulation of undigested substrates and altered intracellular trafficking. Several lysosomal storage diseases show an impaired fusion of lysosomes with the autophagosomes, resulting in the accumulation of polyubiquitinated aggregates of proteins, mitochondria dysfunction, and cell death [94]. NPC1 is an inherited disease, characterized by mutations in the gene encoding the cholesterol transporter NPC1, which is a transmembrane protein localized at the membrane of late endosomes and lysosomes [95]. NPC1 mutations produce the accumulation of unesterified cholesterol, sphingolipids, and gangliosides in the lysosomes that become enlarged [95]. Although NPC1 is ubiquitously expressed, mutations in NPC1 result in selective neuronal damage that induces neurodegeneration, resulting in the primary cause of lethality in NPC1 patients [95]. Similar to AD, NPC1 patients show neurofibrillary tangles, Aβ plaques, and dystrophic neurites [95]. Human embryonic stem cell (hESC) models of NPC1 show induction of autophagy and altered clearance of autophagic proteins, such as LC3-II [96]. Also, these cell models reveal the presence of mitochondrial fragments, suggesting that aberrant autophagy plays a pathological role in NPC1 [97]. In addition, NPC1 is characterized by impaired Ca2+ lysosomal storage and impaired Ca2+ release from the lysosomes, which in turn affect endosomal-lysosomal fusion and trafficking, further enhancing the accumulation of cholesterol, sphingomyelin, and glycosphingolipids [98]. Curcumin treatment improves Ca2+ homeostasis in an NPC1 mice model, contributing to slowing the disease progression [99].

### 4.2. Other Neurological Conditions

Brain ischemia is a disease that shares several pathological characteristics with AD. Both diseases show neuronal cell death in the CA1 region of the hippocampus, cognitive deficit, Aβ, and tau pathology. In addition, brain ischemia is a risk factor for AD pathology [100]. The genes involved in mitophagy increase shortly after brain ischemia [100]. Curcumin has a beneficial effect after stroke damage by modulating Aβ and tau pathology, as described above [100]. On the other hand, Zhang and colleagues showed that curcumin attenuates cerebral ischemia injury in hypoxia and ischemia rat models and oxygen–glucose-deprived PC12 cells by blocking the over-activation of autophagy [101].

HIV-induced neurocognitive disorder (HAND). More than 50% of HIV patients develop HAND, which consists of cognitive, behavioral, and motor decline [102]. Inflammation and microglia activation are involved in HAND [103]. HAND is due to the infiltration across the BBB of peripheral blood mononuclear cells (PBMCs)/macrophages infected by HIV, which secrete viral proteins, such as gp120 and Tat, or show viral replication that infects the resident microglia [104]. In HAND, microglia show a persistent activation due to gp12 and Tat, leading to a chronic inflammation that contributes to neurodegeneration [104]. Reduction of autophagy in microglia leads to a decrement of inflammatory response [105]. Thus, reduction of autophagy in microglia is a therapeutic strategy. Indeed, Chen and colleagues demonstrated that pretreatment with curcumin reduces autophagy in vitro in a gp-120-infected BV2 microglial cell line, reducing the expression of the pro-inflammatory MCP-1 and IL-17 [102]. The authors show that gp-120 induces the expression of LC3 II and Atg5 in BV2 cells. Pretreatment with curcumin inhibited gp120-induced LC3 and Atg5 over-expression by blocking NF-kB translocation to the nucleus [102]. Curcumin inhibited NF-kB by blocking the PI3K-AKT-IKK pathway [102].

## 5. Effect of Curcumin on the Autophagy Pathways in Neuromuscular Diseases

**Figure 4:**
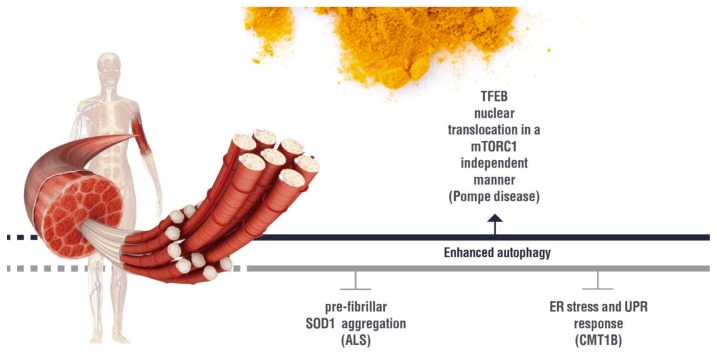
Overview of curcumin effect on the autophagy pathways in neuromuscular diseases.Biological effect of curcumin on the molecules involved in the autophagy regulation in the neuromuscular diseases discussed in the present review. 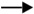 (induction); 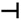 (inhibition). ALS: Amyotrophic lateral sclerosis; ER:Endoplasmic Reticulum; UPR: Unfolded Protein Response; CMT1B: Charcot Marie Tooth 1B.

Recent research has revealed that autophagy is dysregulated in several neuromuscular diseases and can contribute to the disease progression. In some of these diseases, the therapeutic potential of curcumin has been demonstrated. Possible autophagic molecular targets of curcumin in neuromuscular diseases are summarized in Figure 4.

Neuromuscular diseases represent a spectrum of pathologies affecting the peripheral nervous system, including the anterior horn cell, the peripheral nerve, the neuromuscular junction, and the muscle. They are all characterized by impaired muscle function and may also show joint contractures, skeletal alterations, affections of the sensory system (neuropathies), respiratory failure, and dynamic impairments.

Amyotrophic lateral sclerosis (ALS) is a fatal neuromuscular disease due to the progressive loss of upper and lower motor neurons at the spinal or bulbar level [106]. ALS can be divided into (a) familiar, characterized by the presence of inherited mutations in one of the following genes that are causative of this disease: superoxide dismutase 1 (SOD1), VCP, TAR DNA binding protein 43 (TDP-43), ubiquilin 2, C9orf72, and profiling, and (b) sporadic, which account for the 90–95% of the cases [107]. It is noteworthy that pathologic aggregates of TDP-43 occur in 97% of ALS cases [108]. Thus, TDP-43 is a common denominator for the large majority of ALS cases. The enzyme SOD1 converts O2- in H2O2, which is in turn transformed into H2O. Mutations in SOD1 induce ROS production and oxidative stress, and lead to the formation of SOD1 aggregates [109]. SOD1 mutants also alter proteostasis [110]. Oxidative stress produces the accumulation of oxidatively modified macromolecules and damaged cellular organelles. Autophagy is involved in the elimination of these toxic products, protecting cells from oxidative damage by eliminating oxidatively damaged endoplasmic reticulum, mitochondria, peroxisomes, and aggregated proteins [111]. Excessive O- or inhibition of SOD induce autophagy as a protective mechanism [112]. TDP-43 is a ubiquitously expressed and well-conserved DNA/RNA binding protein, which regulates RNA splicing and microRNA formation [113,114,115]. TDP-43 typically shows nuclear localization. However, in ALS, it is localized in the cytoplasm, where it is ubiquitinated and phosphorylated [116]. 4-hydroxy-2-nonenal (HNE), a product of lipid peroxidation, plays a role in TDP-43-mediated pathology by inducing TDP-43 modifications, cytoplasmic localization, and insolubilization [117]. Removal of SOD1 and TDP-43 aggregates by induction of autophagy blocks apoptosis and neuronal loss and slows down ALS progression [118]. There are a few studies that analyze the role of curcumin as a therapeutic agent against ALS. As we described above, the inflammatory response of innate immunity participates in the progression of neurodegeneration. It has been shown that curcumin not only enhances the clearance of misfolded/aggregate SOD1 by the innate immunity cells but also inhibits the inflammatory cascade in these cells [119]. We already mentioned that curcumin inhibits the aggregation of amyloidogenic proteins and peptides and induces their degradation. Indeed, curcumin binds pre-fibrillar SOD1, blocking its further aggregation [120]. A clinical study showed that treatment for 12 months with nanocurcumin together with a pharmacological treatment was well tolerated and also suggested an improvement of survival compared to patients who received only the pharmacological treatment [121].

Desminopathy a myofibrillar myopathy characterized by the presence of sarcoplasmic accumulation of protein aggregates, leading to a degeneration of the myofibrillar apparatus. Desmin is a muscle-specific intermediate filament forming the sarcoplasmic network maintaining the spatial relationship between the contractile apparatus and the other structural elements of the muscle fibers [122]. Mutations in the desmin gene are associated with desminopathy, which disrupts the asset of the contractile apparatus [123] and alters the cytoskeleton, affecting the distribution and function of the mitochondria [124]. The formation of sarcoplasmic aggregates of mutant desmin damages the quality control system of the cell (HSP, the ubiquitin proteasome system UPS, autophagy), leading to the onset of the disease in adult years. Since this disease occurs in adults, it has been hypothesized that a therapy activating the quality-control system could delay the onset of desminopathy. The effect of curcumin was analyzed in vitro in a C2C12 muscle cell line, expressing GFP-Desmin D399Y [125]. The induction of autophagy with PP424 (mTOR inhibitor), detected by an increased level of LC3-II, reduces mutant desmin aggregates [125]. In this cellular system, a treatment combining PP424, antioxidant, and modulators of cell signaling pathways induces autophagy and reduces desmin aggregation efficiently [125].

Charcot Marie tooth 1B (CMT 1B). It is an inherited motor and sensor neuropathy due to mutation of the MPZ gene, which encodes the myelin protein zero that is involved in nerve myelination [126]. Mutant myelin protein zero accumulates in the endoplasmic reticulum (ER) of the Schwann cells, leading to the activation of the unfolded protein response (UPR) [126]. Accumulation of myelin protein zero results in Schwann cell dedifferentiation and subsequent nerve demyelination [126]. Using an in vitro cellular system expressing mutants of myelin protein zero that accumulate in the ER, it has been demonstrated that curcumin releases the ER-retaining mutant MPZ product into the cytosol, inhibiting cellular apoptosis [127]. An R89C mice model of CMT 1B treated either with curcumin dissolved in sesame oil or with phosphatidylcholine curcumin starting at four days old shows amelioration of peripheral neuropathy [126]. The beneficial effect of both treatments is due to reduced ER stress and UPR response as well as induction of Schwann cell differentiation [126].

Charcot Marie tooth 1A (CMT 1A), Dejerine–Sottas neuropathy (DSN), congenital hypomyelinating neuropathy (CHN), and hereditary neuropathy with liability to pressure palsies (HNPP) are in a family of neuropathies resulting from a mutation of the PMP22 gene, which encodes the myelin protein 22 [128]. Mutant myelin protein 22 accumulates in the ER of Schwann cells, leading to impaired myelination and Schwann cell apoptosis. Curcumin treatment in vitro of cells expressing mutant PMP 22 results in the release of myelin protein 22 from the ER to the cytosol and blocks aggregation-induced apoptosis [128]. Oral administration of curcumin to the Trembler-J mice model of CMT 1A strongly ameliorated peripheral neuropathy and Schwann cell apoptosis [128].

Pompe Disease (Glycogen storage disease type II) is a severe, inherited neuromuscular disorder characterized by a deficit of the lysosomal acidα-glucosidase (GAA) [98]. Inside the acidic environment of the lysosomes, GAA is the only enzyme that converts glycogen into glucose. Thus, a deficit of GAA results in glycogen accumulation inside the lysosomes, which become enlarged [98]. This disease shows an extensive spectrum of clinical phenotypes, from a very severe infantile form with cardiopathy and muscle weakness to a less severe form lacking a cardiac phenotype and with a slowly progressive skeletal muscle myopathy that frequently leads to respiratory deficiency [98]. The GAA KO mice model also showed impaired autophagy and mitochondria dysfunction [98]. Altered lysosomes in the muscle of Pompe Disease patients are ubiquitinated and are recruited by the autophagic flux, affecting the cell lysosomal capacity [98]. TFEB and TFE3, which stimulate the expression of genes involved in autophagy and lysosome biogenesis, are a therapeutic target for Pompe Disease [98]. Indeed, promising results have been obtained by over-expressing TFEB for 45 days in the GAA mice model [129]. Since it has been shown that the curcumin analog C1 induces TFEB nuclear translocation in a mTORC1-independent manner [70], we could hypothesize that C1 may be beneficial in slowing the progression of Pompe Disease.

## 6. Conclusions

In conclusion, curcumin has been reported to show versatile bioactivity in modulating the autophagy in neurological and neuromuscular diseases.

Curcumin showed beneficial effects in neuronal cells of diseases characterized by the aggregation of misfolded protein, such as AD, stroke, PD, and ALS. In these disorders, protein aggregates impair the molecular mechanisms of autophagy, leading to neurodegeneration, and curcumin is able not only to inhibit aggregate formations but also to disassemble the aggregates already present. Furthermore, curcumin chelates the metals that facilitate protein aggregation and also enhances the CMA. Several studies demonstrated that in neuronal cells, curcumin restores autophagic impairment caused by aggregate accumulations through modulation of the autophagy signaling pathways. In particular, curcumin can counteract autophagy failure in neuronal cells, reducing mTOR, PI3K, and Akt activation and restoring LAMP-2, beclin, and LC3 I/II levels.

On the other hand, in HAND and ALS diseases, curcumin counteracts the excessive activation of autophagy in innate immunity, lowering chronic inflammation that participates in neurodegeneration (Figure 3), while in NPC1, curcumin restores the Ca2+ homeostasis, ameliorating the function of the autophagic flux. In CMT 1A/B, the function of the Schwann cells (SC) is impaired, leading to demyelination and SC de-differentiation/death. Curcumin targets these cells, inhibiting the ER stress and restoring the UPR (Figure 3).

Few data are available on the effect of curcumin on muscle cells in the context of neuromuscular diseases. However, the literature suggests a beneficial effect of curcumin in this cell type by restoring the autophagy machinery (Figure 4).

We can conclude that curcumin and its synthetic analogs are promising therapeutic agents for neurologic and neuromuscular diseases because of their cell-type specific effects. Curcumin shows a differential activity in different cell types, enhancing autophagy in the cells where autophagy is impaired and inhibiting autophagy in the cell types where it is over-activated. These differential effects converge in preventing/ameliorating the disease progression without producing adverse effects on body physiology.
